# Association of the Platelet‐to‐Neutrophil Ratio With Metabolic Dysfunction‐Associated Steatotic Liver Disease and Liver Fibrosis in U.S. Adults: NHANES 2017–March 2020

**DOI:** 10.1155/ije/5591067

**Published:** 2026-10-13

**Authors:** Yuguan Zhang, Huaibin Zhang, Shanshan Li, Baoyong Zhou, Zhongjun Wu, Hang Yang

**Affiliations:** ^1^ Department of Hepatobiliary Surgery the First Affiliated Hospital of Chongqing Medical University 400016 Chongqing China cqmu.edu.cn; ^2^ Department of Hematology the Second Affiliated Hospital of Chongqing Medical University Chongqing 401336 China cqmu.edu.cn; ^3^ Department of Hepatobiliary Surgery Bishan Hospital of Chongqing Medical University Chongqing 402760 China cqmu.edu.cn; ^4^ Department of Anorectal Surgery Chongqing Traditional Chinese Medicine Hospital Chongqing 400021 China

**Keywords:** liver fibrosis, metabolic dysfunction-associated steatotic liver disease, NHANES, platelet-to-neutrophil ratio

## Abstract

**Background:**

Metabolic dysfunction‐associated steatotic liver disease (MASLD) is a chronic liver disease that is not related to alcohol and is characterized by excessive accumulation of fat within liver cells. As one of the most common chronic liver diseases worldwide, MASLD affects about a quarter of the world’s population and is expected to become a leading indication for liver transplantation by 2030. The disease not only increases the risk of cirrhosis and hepatocellular carcinoma, but also is closely associated with a variety of extra‐hepatic complications such as type 2 diabetes and cardiovascular disease. Although many biomarkers such as N‐terminal type III collagen propeptide (PRO‐C3), enhanced liver fibrosis (ELF), and Fibrosis‐4 Score (FIB‐4) have been used to identify and predict MASLD and metabolic dysfunction‐associated steatohepatitis (MASH), their clinical utility remains limited. Recent studies have shown that neutrophils and platelets play a crucial role in the progression of MASLD. The platelet‐to‐neutrophil ratio (PNR), a novel biomarker, has shown predictive value in ovarian cancer and myocardial infarction. However, PNR has not been fully studied in MASLD. The aim of this study was to investigate the association between PNR and MASLD and to explore its relationship with hepatic steatosis and fibrosis.

**Methods:**

This cross‐sectional study included participants from the National Health and Nutrition Examination Survey (NHANES) 2017–March 2020 pre‐pandemic dataset. Hepatic steatosis was defined as a controlled attenuation parameter (CAP) ≥ 274 dB/m, and MASLD was defined as hepatic steatosis plus at least one cardiometabolic criterion according to the 2023 multisociety steatotic liver disease (SLD) nomenclature. Advanced liver fibrosis was defined as a liver stiffness measurement (LSM) ≥ 8.2 kPa. PNR was calculated as the platelet count divided by the neutrophil count and log2‐transformed (log2‐PNR). Missing covariates were handled by multiple imputation (*m* = 20). Survey‐weighted logistic regression, natural‐spline smoothing, threshold effect analysis, and subgroup analyses were used to evaluate the association between log2‐PNR and MASLD.

**Results:**

A total of 3437 adults aged 18 years and older were included (mean age 47.5 ± 17.5 years; 48.1% male). In the fully adjusted model, higher log2‐PNR was inversely associated with MASLD (OR 0.75 per doubling of PNR, 95% CI 0.63–0.90), and with hepatic steatosis; participants in the highest PNR quartile had lower odds of MASLD than those in the lowest quartile. Threshold effect analysis identified a breakpoint at PNR approximately 38 for MASLD, with a significant nonlinear pattern. Higher log2‐PNR was also inversely associated with advanced fibrosis (OR 0.43, 95% CI 0.33–0.57), with no significant nonlinearity. Subgroup analyses showed that the inverse association was more pronounced in males and non‐Hispanic White participants for MASLD (P for interaction = 0.009 and 0.037, respectively).

**Conclusion:**

Higher log2‐PNR was inversely associated with the cross‐sectional presence of MASLD and hepatic steatosis, and with advanced liver fibrosis, in U.S. adults. Log2‐PNR, an easily obtained inflammatory marker, is independently associated with MASLD. These findings require validation in prospective cohort studies.

## 1. Introduction

Metabolic dysfunction‐associated steatotic liver disease (MASLD) is one of the most common chronic liver diseases, affecting approximately 32.4% of adults worldwide [1]. This is a serious threat to human life and poses a major challenge to the global health care system. Pathologically and clinically, MASLD can cause a wide range of liver lesions, from the initial pathological changes dominated by inflammatory cell infiltration and hepatocyte ballooning to the late changes characterized by liver fibrosis, which can significantly increase the risk of liver cancer in the long run [2, 3]. In addition, MASLD not only causes liver problems, but also is closely related to metabolic disorders such as dyslipidemia, hyperlipidemia, obesity, insulin resistance, diabetes, and hypertension [4–6]. These diseases, along with metabolic disorders, significantly increase the risk of cardiovascular disease and mortality in people with MASLD [7]. Given these knowledge gaps and the limitations of existing biomarkers, further investigation is needed to better understand the factors associated with MASLD and to evaluate the role of easily obtained markers.

Emerging evidence underscores the central role of immune dysregulation in driving the progression of MASLD [8]. Neutrophils in particular have been shown to play a dual role in MASLD pathogenesis: during early stages, neutrophil infiltration triggers hepatic stellate cell activation via the release of neutrophil elastase (NE) and myeloperoxidase (MPO), while in advanced disease, the formation of neutrophil extracellular traps (NETs) creates a CXCL4‐platelet amplification loop that sustains chronic inflammation and hypercoagulability. This suggests that combining neutrophil and platelet parameters offers value in the evaluation of MASLD [9, 10]. PNR has been identified as an independent prognostic factor for ovarian cancer and a predictor of aneurysm rupture [11, 12]. Studies on the neutrophil‐to‐platelet ratio (NPR) revealed that elevated NPR is associated with poor prognosis in myocardial infarction patients, suggesting that platelet–neutrophil imbalances may mirror activation of the inflammatory‐thrombotic axis [13]. Similarly, the progression of MASLD is accompanied by activation of the inflammatory‐thrombotic axis [14, 15]. In MASLD patients and animal models, neutrophil counts change dynamically [16]. The literature suggests that platelet count is an independent predictor of cirrhosis [17, 18]. Although platelet counts are not sensitive during the fibrotic phase of MASLD, their persistent decline may reflect serious impairment of liver function in the end stage of the disease. Therefore, PNR, as a composite indicator integrating inflammation and thrombus status, has theoretical potential in the dynamic monitoring of MASLD.

The National Health and Nutrition Examination Survey (NHANES), conducted by the Centers for Disease Control and Prevention (CDC), employs a multistage stratified probability sampling design to recruit a nationally representative sample of the U.S. civilian population [19]. Because data collection for the 2019–2020 cycle was suspended in March 2020 due to the coronavirus disease 2019 pandemic, the data collected from 2019 to March 2020 were combined with the 2017–2018 cycle to form the nationally representative NHANES 2017–March 2020 pre‐pandemic dataset, with special sample weights provided for analysis. This dataset includes liver ultrasound transient elastography (LUTE), which provides objective measurements of hepatic steatosis and fibrosis.

To fill existing knowledge gaps in the field of MASLD, we conducted a cross‐sectional study using the NHANES 2017–March 2020 pre‐pandemic data to assess the association between PNR and MASLD in the U.S. adult population, and to explore its relationship with hepatic steatosis and advanced liver fibrosis.

## 2. Materials and Methods

### 2.1. Study Participants

Data from the NHANES 2017–March 2020 pre‐pandemic combined‐release dataset were analyzed in this study, which included LUTE to assess liver status. Detailed information about the NHANES study design and dataset can be accessed at https://www.cdc.gov/nchs/nhanes/. Starting from 15,560 participants, the following stepwise exclusion criteria were applied (Figure 1): (1) participants aged below 18 years (*n* = 5867); (2) participants not in the morning fasting subsample (*n* = 5255); (3) missing platelet or neutrophil counts (*n* = 205); (4) missing or invalid transient elastography (*n* = 270); (5) current hepatitis B virus (HBV) infection, defined as a positive hepatitis B surface antigen (*n* = 25); (6) current hepatitis C virus (HCV) infection, defined as a positive HCV RNA (*n* = 47); (7) heavy alcohol consumption (> 5 drinks/day for males and > 4 drinks/day for females) (*n* = 234); and (8) zero survey weight or undeterminable MASLD outcome (*n* = 220). After exclusion, 3437 participants were included in the final analysis. The NCHS Ethics Review Board approved the NHANES protocols (2017–2018: Protocol #2018–01; 2019–2020: continuation of #2018–01), and all participants provided written informed consent [19].

**FIGURE 1:**
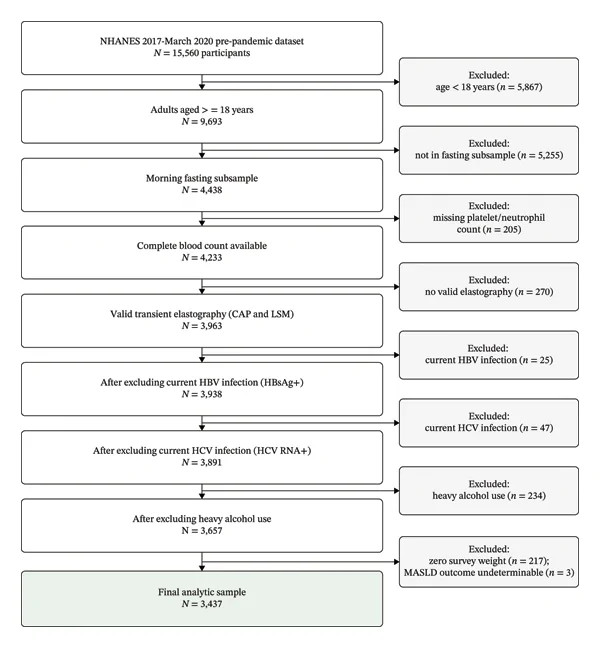
Flowchart of participant selection from the NHANES 2017–March 2020 pre‐pandemic dataset.

### 2.2. Definition of PNR

The Beckman Coulter DxH 800 instrument in the NHANES Mobile Examination Centers (MECs) was used to measure neutrophil and platelet counts, expressed in units of 10^3^ cells per microliter (10^3^ cells/μL). The ratio obtained by dividing platelets by neutrophils is PNR, the primary exposure variable in this analysis. Given the skewed distribution of PNR, all regression models used log2‐transformed PNR (log2‐PNR) as the exposure variable; effect estimates are therefore interpreted per doubling of PNR. PNR may reflect the complex interaction between platelet activation, systemic inflammation, and hepatic fibrosis progression in patients with MASLD, which also indirectly reveals its research value.

### 2.3. Definition of MASLD and Liver Fibrosis

The NHANES MECs utilize FibroScan 502 V2 Touch devices (Echosens, Paris, France) equipped with large or medium probes to perform liver elastography procedures, which are conducted by certified technicians. Valid examination results require fulfillment of three quality control criteria: (1) a minimum 3‐h fasting period prior to assessment, (2) successful completion of at least 10 valid measurements, and (3) an interquartile range‐to‐median ratio (IQR/M) for liver stiffness measurements (LSMs) below 30%. LUTE assessments concurrently quantified LSM and hepatic ultrasound attenuation (controlled attenuation parameter, CAP).

Hepatic steatosis was defined as CAP ≥ 274 dB/m, and participants with LSM ≥ 8.2 kPa were defined as having advanced liver fibrosis [20]. Because CAP identifies hepatic steatosis but cannot by itself establish the etiological classification, MASLD was defined following the 2023 multisociety steatotic liver disease (SLD) nomenclature [21] as hepatic steatosis (CAP ≥ 274 dB/m) plus at least one of five cardiometabolic criteria: (1) body mass index ≥ 25 kg/m^2^ or waist circumference ≥ 94 cm in men/ ≥ 80 cm in women; (2) fasting plasma glucose ≥ 100 mg/dL, hemoglobin A1c ≥ 5.7%, or a diagnosis of or treatment for type 2 diabetes; (3) blood pressure ≥ 130/85 mmHg or antihypertensive treatment; (4) plasma triglycerides ≥ 150 mg/dL or lipid‐lowering treatment; and (5) high‐density lipoprotein cholesterol ≤ 40 mg/dL in men/ ≤ 50 mg/dL in women or lipid‐lowering treatment. Participants with heavy alcohol use were excluded to minimize misclassification of metabolic‐ and‐alcohol‐related liver disease (MetALD) and alcohol‐related liver disease.

### 2.4. Covariates

In order to better clarify the relationship between PNR and MASLD, we included potential covariates that may confound the relationship between the two. These included demographic characteristics (age, sex, race and ethnicity, education level, marital status, and family income‐to‐poverty ratio), lifestyle factors (smoking status, physical activity, and alcohol intake), clinical conditions (hypertension, diabetes, and dyslipidemia), and biochemical markers (alanine aminotransferase [ALT], aspartate aminotransferase [AST], gamma‐glutamyltransferase [GGT], albumin, and total cholesterol). These covariates were measured and investigated by trained healthcare professionals [22]. Operational definitions of all covariates, including the corresponding NHANES variable names, units, and categorization methods, are provided in Supporting Table S1.

### 2.5. Statistical Analysis

All statistical analyses were performed by the authors using NHANES data, incorporating the fasting subsample weights (WTSAFPRP) to adapt to the complex, multistage, clustered survey design. This weighting mechanism ensures national representativeness and corrects for stratification, clustering, and unequal selection probabilities inherent in NHANES data collection. Continuous variables are reported as weighted mean ± standard deviation, while categorical variables are expressed as weighted percentages. Group comparisons across PNR quartiles employed survey‐weighted linear regression or the Rao‐Scott chi‐square test.

Missing covariate values were handled by multiple imputation using chained equations (mice package, *m* = 20 imputations); complete‐case analysis was additionally presented as a sensitivity analysis. Because of multiple imputation, all regression models used the full sample of 3437 participants. To investigate the association between MASLD and PNR, survey‐weighted logistic regression models were performed in four scenarios. Model 1 was unadjusted. Model 2 was adjusted for age, sex, and race/ethnicity. Model 3 (the main model) was further adjusted for education level, marital status, income‐to‐poverty ratio, smoking status, and physical activity. Model 4 additionally adjusted for body mass index, diabetes, hypertension, dyslipidemia, and ALT. Because body mass index, diabetes, hypertension, and dyslipidemia are components of the cardiometabolic criteria used to define MASLD, Model 4 was treated as a sensitivity analysis rather than the primary model. PNR was modeled both as a continuous variable (log2‐PNR, per doubling of PNR) and in quartiles (with the lowest quartile as the reference), with P for trend tested using quartile medians as a continuous variable.

Nonlinear relationships between log2‐PNR and the outcomes were probed using survey‐weighted generalized additive models with natural splines (degrees of freedom = 3), and a Wald test comparing the spline model with a linear model was used to assess nonlinearity. Threshold effects were evaluated with survey‐weighted two‐piecewise logistic regression; the breakpoint was identified by grid search across the 10th–90th percentiles of log2‐PNR and confirmed by likelihood‐ratio testing, with an approximate 95% confidence interval derived from the deviance profile. Subgroup analyses were performed by age, sex, body mass index, race/ethnicity, education level, hypertension, diabetes, dyslipidemia, and smoking status; in each subgroup model, the stratifying variable was removed from the covariate set, and interaction was tested with a pooled Wald test. Subgroup analyses were exploratory. A two‐sided *p*‐value < 0.05 was considered statistically significant. All analyses were performed using *R* version 4.5.1 (http://www.R-project.org, The *R* Foundation) with the survey and mice packages.

## 3. Results

### 3.1. Baseline Characteristics of Participants

A total of 3437 participants were included in the final analysis (Table 1). The weighted mean age of the cohort was 47.5 ± 17.5 years, with 48.1% males. The weighted prevalence of hepatic steatosis (CAP ≥ 274 dB/m) was 41.8%, the weighted prevalence of MASLD was 41.7%, and the weighted prevalence of advanced fibrosis (LSM ≥ 8.2 kPa) was 8.3%. Participants in higher PNR quartiles were younger, had lower BMI, lower CAP and LSM, and lower prevalences of diabetes, hypertension, dyslipidemia, steatosis, and advanced fibrosis (all *p* < 0.05). The unweighted event counts within each PNR quartile are shown in Table 1 and Supporting Table S2.

**TABLE 1:** Baseline characteristics of the study population by PNR quartiles (weighted).

Variable	Overall (*N* = 3437)	Q1 (*n* = 860)	Q2 (*n* = 859)	Q3 (*n* = 859)	Q4 (*n* = 859)	*p*‐value
N (unweighted)	3437	860	859	859	859	
Age (years)	47.5 ± 17.5	51.7 ± 17.7	46.8 ± 17.6	45.8 ± 17.5	45.5 ± 16.4	< 0.001
BMI (kg/m^2^)	29.4 ± 7.2	31.2 ± 7.8	29.8 ± 7.7	28.6 ± 6.5	27.9 ± 6.1	< 0.001
CAP (dB/m)	263.5 ± 62.2	279.3 ± 67.4	265.2 ± 65.4	257.6 ± 56.6	250.5 ± 53.6	< 0.001
LSM (kPa)	5.7 ± 4.2	6.6 ± 5.7	5.6 ± 3.7	5.4 ± 4.3	5.1 ± 2.2	< 0.001
PNR	69.5 ± 30.7	38.7 ± 7.2	57.3 ± 4.5	74.7 ± 6.0	111.8 ± 29.5	< 0.001
log_2_‐PNR	6.0 ± 0.6	5.2 ± 0.3	5.8 ± 0.1	6.2 ± 0.1	6.8 ± 0.3	< 0.001
Sex, *n* (%)						< 0.001
Female	51.9	41.9	53.5	56.1	56.6	
Male	48.1	58.1	46.5	43.9	43.4	
Race/ethnicity, *n* (%)						< 0.001
Mexican American	8.6	8.9	10.3	8.4	6.8	
Other Hispanic	7	8.3	6.5	5.8	7.1	
Non‐Hispanic White	62.8	66.8	64.7	62.7	56.1	
Non‐Hispanic Black	11.3	6.4	8.6	11.5	19.6	
Non‐Hispanic Asian	6	4.8	6.1	6.2	7	
Other/multiracial	4.3	4.7	3.8	5.3	3.4	
Education level, n (%)						0.010
< 9th grade	3.6	4.2	3.3	4.4	2.3	
9–11th grade	6.9	8.3	8.6	5.8	4.7	
High school/GED	25.4	29.6	26.3	23.7	21.5	
Some college/AA	30.1	31.2	28.9	28.8	31.5	
College+	34	26.7	32.8	37.3	40	
Marital status, *n* (%)						0.021
Not married	37.5	38.6	34.4	34	43.3	
Married/living with partner	62.5	61.4	65.6	66	56.7	
Smoking status, *n* (%)						0.003
Never	59.6	51.7	57.6	63.2	66.6	
Current	13.9	18.5	14.7	14	7.6	
Former	26.6	29.8	27.6	22.7	25.9	
Diabetes, *n* (%)						< 0.001
No	84.1	74.3	81.7	88.6	92.9	
Yes	15.9	25.7	18.3	11.4	7.1	
Hypertension, *n* (%)						< 0.001
No	68.8	56.4	69	74.6	76.2	
Yes	31.2	43.6	31	25.4	23.8	
Dyslipidemia, *n* (%)						< 0.001
No	49.3	39.4	43.5	56.9	58.7	
Yes	50.7	60.6	56.5	43.1	41.3	
Physically active, *n* (%)						0.747
No	74.7	75.3	76.1	74.6	72.6	
Yes	25.3	24.7	23.9	25.4	27.4	
Steatosis, *n* (%)						< 0.001
No	58.2	49.4	56.6	61.2	66.6	
Yes	41.8	50.6	43.4	38.8	33.4	
MASLD, *n* (%)						< 0.001
No	58.3	49.4	56.6	61.5	66.9	
Yes	41.7	50.6	43.4	38.5	33.1	
Advanced fibrosis, *n* (%)						< 0.001
No	91.7	85.3	90.4	95.2	96.7	
Yes	8.3	14.7	9.6	4.8	3.3	

*Note:* Values are weighted mean ± SD for continuous variables and weighted percentage for categorical variables. *p*‐values from survey‐weighted linear regression (continuous) or Rao–Scott chi‐square test (categorical).

Abbreviations: CAP, controlled attenuation parameter; LSM, liver stiffness measurement; PIR, income‐to‐poverty ratio; PNR, platelet‐to‐neutrophil ratio.

### 3.2. Association of log2‐PNR With MASLD and Advanced Fibrosis

In survey‐weighted logistic regression models pooled over 20 multiple imputations, higher log2‐PNR was inversely associated with MASLD (Table 2; Figure 2). Compared with the unadjusted model (Model 1: OR 0.64, 95% CI 0.54–0.76), the association remained significant after adjustment for age, sex, and race/ethnicity (Model 2: OR 0.74, 95% CI 0.62–0.88) and in the main fully adjusted model (Model 3: OR 0.75, 95% CI 0.63–0.90; P for trend < 0.001). In the sensitivity model additionally adjusting for body mass index, diabetes, hypertension, dyslipidemia, and ALT (Model 4), the association was attenuated and no longer statistically significant (OR 1.17, 95% CI 0.93–1.49). In quartile analyses, participants in the highest PNR quartile had 38% lower odds of MASLD than those in the lowest quartile in Model 3 (OR 0.62, 95% CI 0.48–0.81; P for trend < 0.001).

**TABLE 2:** Association of log2‐PNR with MASLD and advanced fibrosis: multiple imputation analysis.

Variable	Model 1	Model 2	Model 3	Model 4
MASLD				
log2‐PNR (per doubling of PNR)	0.64 (0.54–0.76)	0.74 (0.62–0.88)	0.75 (0.63–0.90)	1.17 (0.93–1.49)
*p*‐value	< 0.001	< 0.001	0.002	0.187
PNR quartiles (reference: Q1)				
Q1	1.00 (reference)	1.00 (reference)	1.00 (reference)	1.00 (reference)
Q2	0.75 (0.55–1.03)	0.87 (0.63–1.19)	0.85 (0.62–1.16)	1.07 (0.75–1.52)
Q3	0.61 (0.46–0.80)	0.74 (0.55–0.99)	0.73 (0.54–0.98)	1.23 (0.82–1.84)
Q4	0.48 (0.38–0.62)	0.60 (0.46–0.78)	0.62 (0.48–0.81)	1.27 (0.91–1.79)
P for trend	< 0.001	< 0.001	< 0.001	0.131
Advanced fibrosis				
log2‐PNR (per doubling of PNR)	0.40 (0.31–0.51)	0.43 (0.33–0.57)	0.43 (0.33–0.57)	0.68 (0.49–0.94)
*p*‐value	< 0.001	< 0.001	< 0.001	0.020
PNR quartiles (reference: Q1)				
Q1	1.00 (reference)	1.00 (reference)	1.00 (reference)	1.00 (reference)
Q2	0.62 (0.42–0.90)	0.67 (0.44–1.02)	0.67 (0.44–1.01)	0.84 (0.51–1.40)
Q3	0.29 (0.17–0.49)	0.33 (0.20–0.54)	0.33 (0.20–0.55)	0.57 (0.32–1.01)
Q4	0.20 (0.12–0.33)	0.22 (0.13–0.39)	0.23 (0.13–0.39)	0.51 (0.28–0.94)
P for trend	< 0.001	< 0.001	< 0.001	0.016

*Note:* Values are odds ratios (95% confidence intervals) from survey‐weighted logistic regression pooled over 20 multiple imputations using Rubin’s rules. Model 1: unadjusted. Model 2: adjusted for age, sex, and race/ethnicity. Model 3 (main model): further adjusted for education, marital status, income‐to‐poverty ratio, smoking, and physical activity. Model 4 (sensitivity): additionally adjusted for body mass index, diabetes, hypertension, dyslipidemia, and ALT. All models use the same 3437 participants.

**FIGURE 2:**
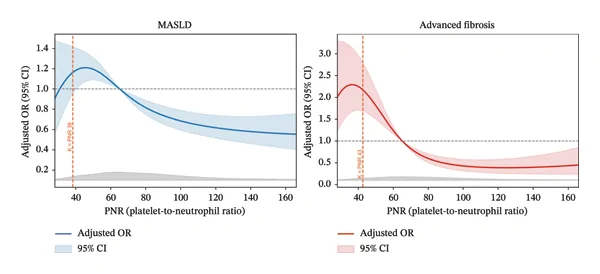
Adjusted dose–response relationship between PNR and MASLD (left) and advanced fibrosis (right). Solid line, adjusted odds ratio; shaded area, 95% confidence interval; dashed vertical line, identified breakpoint; gray area at the bottom, distribution of PNR. All analyses pooled over 20 multiple imputations.

Higher log2‐PNR was also inversely associated with advanced fibrosis (Table 2). The odds ratio per doubling of PNR was 0.40 (95% CI 0.31–0.51) in Model 1 and 0.43 (95% CI 0.33–0.57) in the main adjusted model (Model 3). In the sensitivity model (Model 4), the association was attenuated (OR 0.68, 95% CI 0.49–0.94). Complete‐case analyses yielded consistent point estimates, with the association similarly attenuated in Model 4 in both analyses (Supporting Table S3).

### 3.3. Threshold Effect Analysis

Smooth curve fitting and threshold effect analysis revealed a nonlinear relationship between log2‐PNR and MASLD (nonlinearity test *p* = 0.046; Table 3). Two‐piecewise regression identified a breakpoint at PNR approximately 38 (log2‐PNR 5.26; approximate 95% confidence interval 38–41). Below the breakpoint, which comprised only about 10% of participants, the association appeared positive but was estimated from sparse data with a wide confidence interval (OR 4.07, 95% CI 1.60–10.41); above the breakpoint, which included approximately 90% of the cohort, higher log2‐PNR was robustly associated with lower odds of MASLD (OR 0.60, 95% CI 0.51–0.70; bend test *p* < 0.001).

**TABLE 3:** Threshold effect analysis of log2‐PNR on MASLD and advanced fibrosis.

Outcome	Breakpoint K (PNR)	OR below K (95% CI)	OR above K (95% CI)	Bend *p*‐value	Nonlinearity *p*‐value
MASLD	38.3 (log2‐PNR 5.26)	4.07 (1.60–10.41)	0.60 (0.51–0.70)	< 0.001	0.046
Advanced fibrosis	42.6 (log2‐PNR 5.41)	1.54 (0.74–3.22)	0.27 (0.17–0.42)	< 0.001	0.391

*Note:* The breakpoint was identified by grid search in survey‐weighted two‐piecewise logistic regression and confirmed by likelihood‐ratio testing; approximate 95% confidence intervals for K were PNR 38–41 (MASLD) and 38–51 (advanced fibrosis). ORs below/above K are per doubling of PNR, pooled over 20 multiple imputations. The bend *p*‐value tests whether the slope above K differs from the slope below K; nonlinearity was assessed by a Wald test comparing linear versus natural‐spline (df = 3) models.

For advanced fibrosis, no significant nonlinearity was detected (*p* = 0.391), and the inverse association was monotonic across the observed range of log2‐PNR; the breakpoint identified by grid search (PNR approximately 43) was therefore not interpreted as a threshold (Table 3).

### 3.4. Subgroup Analyses

Subgroup analyses are presented in Table 4 and Figure 3. For MASLD, the inverse association was more pronounced in males (OR 0.57, 95% CI 0.45–0.73) than in females (OR 0.95, 95% CI 0.75–1.19; P for interaction = 0.009), and in non‐Hispanic White participants (OR 0.68, 95% CI 0.52–0.90) than in other racial/ethnic groups (P for interaction = 0.037). For advanced fibrosis, the inverse association appeared more pronounced in participants aged < 60 years (OR 0.37, 95% CI 0.27–0.50) than in those aged ≥ 60 years (OR 0.50, 95% CI 0.34–0.74; P for interaction = 0.043). No statistically significant evidence of effect modification was observed for the remaining subgroups. These subgroup analyses were exploratory and should be interpreted with caution given the multiplicity of comparisons.

**TABLE 4:** Subgroup analyses of the association between log2‐PNR and MASLD and advanced fibrosis.

Subgroup	OR (95% CI)	*p*‐value	P for interaction
MASLD			
Age			0.738
< 60	0.75 (0.63–0.90)	0.002	
≥ 60	0.62 (0.43–0.90)	0.013	
Sex			0.009
Female	0.95 (0.75–1.19)	0.649	
Male	0.57 (0.45–0.73)	< 0.001	
BMI			0.545
< 30	1.02 (0.74–1.42)	0.888	
≥ 30	0.89 (0.67–1.18)	0.405	
Race/ethnicity			0.037
Hispanic	0.85 (0.65–1.12)	0.258	
Non‐Hispanic Black	0.97 (0.74–1.28)	0.853	
Non‐Hispanic White	0.68 (0.52–0.90)	0.007	
Other	0.74 (0.50–1.11)	0.142	
Education			0.763
< = High school	0.72 (0.50–1.04)	0.082	
> High school	0.72 (0.60–0.86)	< 0.001	
High school	0.79 (0.54–1.15)	0.218	
Hypertension			0.236
No	0.91 (0.72–1.14)	0.414	
Yes	0.63 (0.42–0.93)	0.019	
Diabetes			0.538
No	0.88 (0.73–1.05)	0.154	
Yes	0.58 (0.35–0.94)	0.028	
Dyslipidemia			0.071
No	0.97 (0.76–1.25)	0.838	
Yes	0.72 (0.59–0.88)	0.001	
Smoking status			0.057
Current	1.33 (0.80–2.21)	0.265	
Former	0.57 (0.38–0.86)	0.007	
Never	0.73 (0.59–0.90)	0.004	
Advanced fibrosis			
Age			0.043
< 60	0.37 (0.27–0.50)	< 0.001	
≥ 60	0.50 (0.34–0.74)	< 0.001	
Sex			0.037
Female	0.36 (0.24–0.54)	< 0.001	
Male	0.49 (0.34–0.69)	< 0.001	
BMI			0.737
< 30	0.54 (0.36–0.81)	0.003	
≥ 30	0.44 (0.30–0.65)	< 0.001	
Race/ethnicity			0.474
Hispanic	0.38 (0.18–0.79)	0.010	
Non‐Hispanic Black	0.63 (0.40–0.97)	0.035	
Non‐Hispanic White	0.42 (0.29–0.62)	< 0.001	
Other	0.34 (0.19–0.60)	< 0.001	
Education			0.405
< = High school	0.28 (0.15–0.53)	< 0.001	
> High school	0.44 (0.32–0.60)	< 0.001	
High school	0.46 (0.28–0.75)	0.002	
Hypertension			0.418
No	0.41 (0.28–0.61)	< 0.001	
Yes	0.46 (0.31–0.69)	< 0.001	
Diabetes			0.136
No	0.46 (0.32–0.66)	< 0.001	
Yes	0.63 (0.40–0.99)	0.043	
Dyslipidemia			0.905
No	0.47 (0.30–0.75)	0.002	
Yes	0.44 (0.31–0.63)	< 0.001	
Smoking status			0.838
Current	0.40 (0.21–0.76)	0.005	
Former	0.36 (0.21–0.61)	< 0.001	
Never	0.48 (0.33–0.71)	< 0.001	

*Note:* Values are odds ratios (95% confidence intervals) per doubling of PNR from survey‐weighted logistic regression pooled over multiple imputations, adjusted for the Model 3 covariates with the stratifying variable removed from the covariate set. P for interaction from a pooled Wald test. Subgroup analyses are exploratory.

**FIGURE 3:**
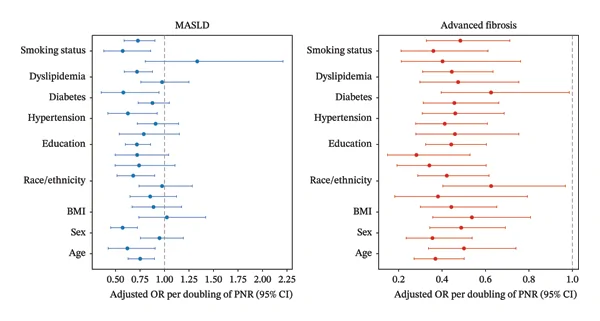
Subgroup analyses of the association between PNR and MASLD (left) and advanced fibrosis (right). Points and error bars, adjusted odds ratio per doubling of PNR with 95% confidence interval; stratifying variable removed from the covariate set; analyses are exploratory.

## 4. Discussion

As one of the major challenges facing the global health care system, MASLD urgently needs new indicators to dynamically evaluate its prevention and prognosis. As a composite indicator of inflammation and thrombosis [23], the evidence supporting the role of PNR in the evaluation of MASLD remains limited. Therefore, evaluating the role of this indicator in MASLD may provide new insights into the management of patients with MASLD.

Our study reveals a significant inverse association between log2‐PNR and MASLD in the main adjusted model, with each doubling of PNR associated with a 25% lower odds of MASLD (OR 0.75, 95% CI 0.63–0.90). Participants in the highest PNR quartile had 38% lower odds of MASLD than those in the lowest quartile. This finding underscores the potential of PNR as a readily available composite marker that is independently associated with MASLD, offering a novel perspective on the interplay between inflammation and liver pathology in MASLD. This is consistent with recent findings that MASLD progression is associated with inflammation [23, 24]. Similarly, higher log2‐PNR was inversely associated with advanced fibrosis in the main models (OR 0.43, 95% CI 0.33–0.57), whereas the association was attenuated in the sensitivity model additionally adjusting for metabolic covariates (OR 0.68, 95% CI 0.49–0.94).

PNR may reflect the dynamic balance between inflammation and thrombosis. Neutrophils are implicated in the pathogenesis of MASLD, with their activation leading to the release of NE and MPO, which in turn trigger hepatic stellate cell activation and liver fibrosis [25, 26]. During this process, platelets participate in inflammation and thrombosis by forming platelet–neutrophil aggregates [14, 24]. In the present study, higher PNR was associated with lower odds of MASLD; because neutrophil‐driven inflammation is implicated in hepatic steatosis, a relatively lower neutrophil burden, as reflected by a higher PNR, may correspond to less metabolic inflammation and, consequently, less steatosis [27]. By integrating these two cellular components, PNR may provide a more comprehensive reflection of the inflammatory and thrombotic milieu relevant to MASLD. Analogously, other composite ratios of inflammatory cells, such as combined neutrophil‐to‐lymphocyte and platelet‐to‐lymphocyte ratios, have shown prognostic value in other diseases [28].

Notably, our analysis uncovers a nonlinear relationship between log2‐PNR and MASLD risk (nonlinearity test *p* = 0.046). Two‐piecewise regression identified a breakpoint at PNR approximately 38 (log2‐PNR 5.26). Below the breakpoint, a range comprising only about 10% of participants, the association appeared positive but was estimated from sparse data with a wide confidence interval; above the breakpoint, which includes approximately 90% of the cohort, higher PNR was robustly associated with lower odds of MASLD (OR 0.60, 95% CI 0.51–0.70). This pattern suggests that the inverse association of PNR with MASLD predominates across the range in which most individuals fall. For advanced fibrosis, no significant nonlinearity was detected (*p* = 0.391), and the inverse association was monotonic. A similar threshold phenomenon has been reported for PNR in other metabolic conditions [29]. Future investigations could explore the biological underpinnings of this threshold effect, potentially shedding light on the differential roles of platelets and neutrophils in MASLD pathogenesis at varying stages of disease progression.

Subgroup analyses were conducted to assess the consistency of the PNR–MASLD association across diverse population strata. For MASLD, the inverse association was more pronounced in males (OR 0.57, 95% CI 0.45–0.73) than in females (OR 0.95, 95% CI 0.75–1.19; P for interaction = 0.009), and in non‐Hispanic White participants (OR 0.68, 95% CI 0.52–0.90) than in other racial/ethnic groups (P for interaction = 0.037). For advanced fibrosis, the association appeared more pronounced in participants aged < 60 years than in those aged ≥ 60 years (P for interaction = 0.043). These findings should be interpreted cautiously given the exploratory nature of subgroup analyses and the multiplicity of comparisons; they warrant further exploration in larger, more diverse cohorts to determine whether sex, age, and race/ethnicity modify the PNR–MASLD relationship.

The biological plausibility underlying the observed association between PNR and prevalent MASLD may be rooted in the intertwined roles of platelets and neutrophils in hepatic inflammation, metabolic dysregulation, and fibrogenesis. While neutrophils are classically viewed as pro‐inflammatory effectors that infiltrate the liver during steatohepatitis and release NETs, MPO, and elastase to promote hepatocyte injury and stellate cell activation [9, 16], platelets have emerged as active modulators—not merely bystanders—in the pathogenesis of fatty liver disease [23, 24].

It has been hypothesized, based on preclinical and clinical evidence, that platelets adhere to sinusoidal endothelial cells in steatotic livers, facilitating leukocyte recruitment and amplifying local inflammation through the release of serotonin, CD40 ligand, and TGF‐β, and that platelet‐derived mediators can activate hepatic stellate cells, promoting collagen deposition even in the absence of overt necroinflammation [23, 24]. We emphasize, however, that our study did not measure platelet activation markers, platelet–neutrophil aggregates, NETs, MPO, NE, cytokines, or coagulation markers; therefore, all mechanistic interpretations presented here are hypotheses rather than direct findings of this study. Moreover, the same PNR value can arise from different combinations of platelet and neutrophil counts, which should be considered when interpreting PNR as a composite marker.

Conversely, in advanced MASLD or cirrhosis, progressive portal hypertension with splenic sequestration leads to thrombocytopenia [17], thereby lowering the numerator of PNR; hypersplenism may additionally reduce circulating neutrophil counts (cirrhosis‐associated neutropenia), and chronic gut‐derived bacterial translocation with endotoxemia sustains systemic immune activation [30]. In advanced disease, PNR is therefore shaped by these cirrhosis‐related hematological derangements rather than reflecting the metabolic‐inflammatory process alone, and cannot be interpreted as a simple marker of hepatic inflammation.

FIB‐4 and MASLD Fibrosis Score (NFS) are established noninvasive markers that are widely used to predict liver fibrosis in patients with MASLD [28, 31]. While these scores combine multiple biochemical parameters (such as age, platelet count, AST, and ALT levels), they may not fully capture the dynamic interaction between inflammation and thrombosis that PNR reflects. This study found that PNR was inversely associated with MASLD, with a threshold effect, suggesting that the influence of PNR may vary with its value range and providing a new perspective for understanding the pathological mechanism of MASLD. However, traditional markers (such as FIB‐4 and NFS) are usually linear or log‐linear with liver fibrosis [32–34], which may be difficult to capture the complex dynamics of MASLD progression. Each marker has its unique advantages and limitations, and future studies may explore the combined application of PNR with FIB‐4, NFS, etc., to more comprehensively evaluate the progression and treatment response of MASLD.

## 5. Strengths and Limitations

Our research has several advantages. First, to our knowledge, this is among the first studies to link PNR with MASLD based on a nationally representative sample, providing new ideas for the evaluation of MASLD. Second, we applied rigorous statistical methods, including survey‐weighted regression accounting for the complex sampling design, multiple imputation for missing covariates, and a trace‐back validation confirming that every value in the analysis cohort matched the original NHANES source files, which enhances the reliability of the findings.

Although we provide evidence for a relationship between MASLD and PNR, our study has several limitations that are worth discussing. As a cross‐sectional analysis, it cannot establish causality between PNR and MASLD. Longitudinal studies are needed to elucidate the temporal relationship and potential causal pathways linking PNR to MASLD development and progression.

While we adjusted for multiple confounders, residual confounding may still be present, for example, detailed information on dietary habits, which may contribute to systemic low‐grade inflammation [35], physical activity levels, and other environmental factors was not available in the NHANES dataset. Future research should aim to capture a broader array of potential confounders to enhance the precision and generalizability of findings.

This study focused primarily on the association between PNR and MASLD and did not delve into the underlying biological mechanisms. Future investigations could employ animal models and in vitro experiments to dissect the molecular pathways through which PNR influences MASLD pathogenesis. Such mechanistic studies could identify novel therapeutic targets and inform the development of personalized interventions for MASLD management.

In addition, several other limitations should be acknowledged. First, hepatic steatosis and fibrosis were assessed by CAP and LSM thresholds rather than liver biopsy, and potential outcome misclassification cannot be excluded. Second, PNR was measured at a single time point, and acute infection, inflammation, or medications that affect platelet or neutrophil counts may influence PNR. Third, despite multiple imputation, residual confounding from unmeasured factors may remain. Fourth, uncertainty inherent to the complex survey design and weighting may affect the estimates. Fifth, observations at extreme PNR values were sparse, which limits the precision of estimates in those ranges. Finally, as a cross‐sectional study, we could not assess the incremental predictive value of PNR beyond established markers, and reverse causality and temporal ordering cannot be established.

## 6. Conclusion

This study demonstrated that higher log2‐PNR was inversely associated with the presence of MASLD and hepatic steatosis in U.S. adults, whereas its association with advanced liver fibrosis was attenuated after accounting for metabolic covariates. These findings suggest that PNR is a readily available composite marker that is independently associated with MASLD; however, further prospective cohort studies are needed to validate these findings and to clarify the temporal relationship.

## Author Contributions

Yuguan Zhang: designed the study, collected the data, and performed preliminary data analysis.

Huaibin Zhang: conducted statistical analysis and interpreted the results.

Shanshan Li: reviewed the data and ensured quality control.

Zhongjun Wu: coordinated the project and allocated resources.

Baoyong Zhou: discussed the results and revised the manuscript.

Hang Yang: finalized the review and submitted the manuscript.

## Funding

This study was supported by the National Natural Science Foundation of China (No. 82400772), Chongqing Natural Science Foundation (CSTB2024NSCQ‐MSX0808), Chongqing science and health joint project (2024GGXM005), and Chongqing Natural Science Foundation (CSTB2023NSCQ‐BHX0131).

## Ethics Statement

In compliance with the Declaration of Helsinki, every NHANES protocol was approved by the Ethics Review Board of the National Center for Health Statistics (NHANES 2017–2018: Protocol #2018–01; NHANES 2019–March 2020: continuation of Protocol #2018–01). All participants provided written informed consent. The present study used publicly available, de‐identified data; our institutional review board determined that additional approval was not required for this secondary analysis.

## Consent

The authors have nothing to report.

## Conflicts of Interest

The authors declare no conflicts of interest.

## Supporting Information

Additional supporting information can be found online in the Supporting Information section.

## Supporting information


**Supporting Information** Supporting Table S1 lists the operational definitions and NHANES variable codes for all covariates, the exposure, and the outcomes. Supporting Table S2 reports unweighted event counts together with weighted numbers of participants, events, and prevalence across quartiles of the platelet‐to‐neutrophil ratio. Supporting Table S3 presents the complete‐case sensitivity analysis restricted to participants with complete covariate data (*n* = 2780), and Supporting Table S4 presents the sensitivity analysis applying the stricter alcohol‐exclusion criterion of the 2023 SLD nomenclature (*n* = 2432). All supporting analyses used the same survey‐weighted, multiple‐imputation framework as the primary analysis.

## Data Availability

The survey results are accessible to academics and data users worldwide over the Internet; for more information, see the NHANES website at http://www.cdc.gov/nchs/nhanes.

## References

[1] Quek J. , Chan K. E. , Wong Z. Y. et al., Global Prevalence of Non-Alcoholic Fatty Liver Disease and Non-Alcoholic Steatohepatitis in the Overweight and Obese Population: A Systematic Review and Meta-Analysis, Lancet Gastroenterology & Hepatology. (2023) 8, no. 1, 20–30, 10.1016/s2468-1253(22)00317-x. 36400097

[2] Yahoo N. , Dudek M. , Knolle P. , and Heikenwälder M. , Role of Immune Responses in the Development of NAFLD-Associated Liver Cancer and Prospects for Therapeutic Modulation, Journal of Hepatology. (2023) 79, no. 2, 538–551, 10.1016/j.jhep.2023.02.033. 36893854

[3] Powell E. E. , Wong V. W. , and Rinella M. , Non-Alcoholic Fatty Liver Disease, Lancet. (2021) 397, no. 10290, 2212–2224, 10.1016/s0140-6736(20)32511-3. 33894145

[4] Byrne C. D. and Targher G. , NAFLD: A Multisystem Disease, Journal of Hepatology. (2015) 62, no. 1, S47–S64, 10.1016/j.jhep.2014.12.012. 25920090

[5] Targher G. , Corey K. E. , Byrne C. D. , and Roden M. , The Complex Link Between NAFLD and Type 2 Diabetes Mellitus-Mechanisms and Treatments, Nature Reviews Gastroenterology & Hepatology. (2021) 18, no. 9, 599–612, 10.1038/s41575-021-00448-y. 33972770

[6] Mager D. R. , Mazurak V. , Rodriguez‐Dimitrescu C. et al., A Meal High in Saturated Fat Evokes Postprandial Dyslipemia, Hyperinsulinemia, and Altered Lipoprotein Expression in Obese Children With and Without Nonalcoholic Fatty Liver Disease, JPEN-Journal of Parenteral and Enteral Nutrition. (2013) 37, no. 4, 517–528, 10.1177/0148607112467820. 23223552

[7] Duell P. B. , Welty F. K. , Miller M. et al., Nonalcoholic Fatty Liver Disease and Cardiovascular Risk: A Scientific Statement From the American Heart Association, Arteriosclerosis, Thrombosis, and Vascular Biology. (2022) 42, no. 6, e168–e185, 10.1161/atv.0000000000000153. 35418240

[8] Huby T. and Gautier E. L. , Immune Cell-Mediated Features of Non-Alcoholic Steatohepatitis, Nature Reviews Immunology. (2022) 22, no. 7, 429–443, 10.1038/s41577-021-00639-3. PMC8570243 34741169

[9] Herrero-Cervera A. , Soehnlein O. , and Kenne E. , Neutrophils in Chronic Inflammatory Diseases, Cellular and Molecular Immunology. (2022) 19, no. 2, 177–191, 10.1038/s41423-021-00832-3. PMC8803838 35039631

[10] Hu Z. B. , Zhong Q. Q. , Lu Z. X. , and Zhu F. , Association of Platelet-to-White Blood Cell Ratio and Platelet-to-Neutrophil Ratio With the Risk of Fatal Stroke Occurrence in Middle-Aged to Older Chinese, BMC Geriatrics. (2022) 22, no. 1, 10.1186/s12877-022-03134-z. PMC9112464 35581556

[11] Yun S. , Jun Yi H. , Hoon Lee D. , and Hoon Sung J. , Clinical Significance of Platelet to Neutrophil Ratio and Platelet to Lymphocyte Ratio in Patients With Aneurysmal Subarachnoid Hemorrhage, Journal of Clinical Neuroscience. (2021) 92, 49–54, 10.1016/j.jocn.2021.07.036. 34509261

[12] Song Q. , Xu S. X. , Wu J. Z. et al., The Preoperative Platelet to Neutrophil Ratio and Lymphocyte to Monocyte Ratio are Superior Prognostic Indicators Compared With Other Inflammatory Biomarkers in Ovarian Cancer, Frontiers in Immunology. (2023) 14, 10.3389/fimmu.2023.1177403. PMC10347525 37457691

[13] Liao J. , Xiong F. , Chen H. et al., Neutrophil to Platelet Ratio Predicts In-Hospital Mortality in Patients With Acute Myocardial Infarction, Internal and Emergency Medicine. (2025) 20, no. 3, 723–731, 10.1007/s11739-025-03859-w. 39838258

[14] Peiseler M. , Schwabe R. , Hampe J. , Kubes P. , Heikenwälder M. , and Tacke F. , Immune Mechanisms Linking Metabolic Injury to Inflammation and Fibrosis in Fatty Liver Disease-Novel Insights Into Cellular Communication Circuits, Journal of Hepatology. (2022) 77, no. 4, 1136–1160, 10.1016/j.jhep.2022.06.012. 35750137

[15] Calès P. , Lainé F. , Boursier J. et al., Comparison of Blood Tests for Liver Fibrosis Specific or Not to NAFLD, Journal of Hepatology. (2009) 50, no. 1, 165–173, 10.1016/j.jhep.2008.07.035. 18977552

[16] Hou X. , Yin S. , Ren R. et al., Myeloid-Cell-Specific IL-6 Signaling Promotes MicroRNA-223-Enriched Exosome Production to Attenuate NAFLD-Associated Fibrosis, Hepatology. (2021) 74, no. 1, 116–132, 10.1002/hep.31658. PMC8141545 33236445

[17] Lisman T. , Caldwell S. H. , and Intagliata N. M. , Haemostatic Alterations and Management of Haemostasis in Patients With Cirrhosis, Journal of Hepatology. (2022) 76, no. 6, 1291–1305, 10.1016/j.jhep.2021.11.004. 35589251

[18] Kozumi K. , Kodama T. , Murai H. et al., Transcriptomics Identify Thrombospondin-2 as a Biomarker for NASH and Advanced Liver Fibrosis, Hepatology. (2021) 74, no. 5, 2452–2466, 10.1002/hep.31995. PMC8596693 34105780

[19] Lloyd-Jones D. M. , Ning H. , Labarthe D. et al., Status of Cardiovascular Health in US Adults and Children Using the American Heart Association’s New Life’s Essential 8 Metrics: Prevalence Estimates From the National Health and Nutrition Examination Survey (NHANES), 2013 Through 2018, Circulation. (2022) 146, no. 11, 822–835, 10.1161/circulationaha.122.060911. 35766033

[20] Yang B. , Gong M. , Zhu X. et al., Correlation Between Liver Fibrosis in Non-Alcoholic Fatty Liver Disease and Insulin Resistance Indicators: A Cross-Sectional Study From NHANES 2017-2020, Frontiers in Endocrinology. (2025) 16, 10.3389/fendo.2025.1514093. PMC11825334 39959621

[21] Rinella M. E. , Lazarus J. V. , Ratziu V. et al., A Multisociety Delphi Consensus Statement on New Fatty Liver Disease Nomenclature, Journal of Hepatology. (2023) 79, no. 6, 1542–1556, 10.1016/j.jhep.2023.06.003. 37364790

[22] Ye M. , He Y. , Xia Y. et al., Association Between Serum Zinc and Non-Alcoholic Fatty Liver Disease and Advanced Liver Fibrosis: NHANES 2011-2016, Biological Trace Element Research. (2025) 203, no. 3, 1305–1316, 10.1007/s12011-024-04261-x. 38861177

[23] Miele L. , Alberelli M. A. , Martini M. et al., Nonalcoholic Fatty Liver Disease (NAFLD) Severity is Associated to a Nonhemostatic Contribution and Proinflammatory Phenotype of Platelets, Translational Research. (2021) 231, 24–38, 10.1016/j.trsl.2020.11.003. 33171266

[24] Malehmir M. , Pfister D. , Gallage S. et al., Platelet GPIbα is a Mediator and Potential Interventional Target for NASH and Subsequent Liver Cancer, Nature Medicine. (2019) 25, no. 4, 641–655, 10.1038/s41591-019-0379-5. PMC12452109 30936549

[25] Ye Q. , Liu Y. , Zhang G. et al., Deficiency of Gluconeogenic Enzyme PCK1 Promotes Metabolic-Associated Fatty Liver Disease Through PI3K/AKT/PDGF Axis Activation in Male Mice, Nature Communications. (2023) 14, no. 1, 10.1038/s41467-023-37142-3. PMC10015095 36918564

[26] Tacke F. , Puengel T. , Loomba R. , and Friedman S. L. , An Integrated View of Anti-Inflammatory and Antifibrotic Targets for the Treatment of NASH, Journal of Hepatology. (2023) 79, no. 2, 552–566, 10.1016/j.jhep.2023.03.038. PMC12164378 37061196

[27] Shrestha S. , Jeon J. H. , and Hong C. W. , Neutrophils in MASLD and MASH, BMB Reports. (2025) 58, no. 3, 116–123, 10.5483/BMBRep.2024-0058. PMC11955729 39757200

[28] Lee J. , Vali Y. , Boursier J. et al., Prognostic Accuracy of FIB-4, NAFLD Fibrosis Score and APRI for NAFLD-Related Events: A Systematic Review, Liver International. (2021) 41, no. 2, 261–270, 10.1111/liv.14669. PMC7898346 32946642

[29] Sun H. , Li Y. , Liu S. , Pan C. , Li D. , and Zhou X. , The Diagnostic Value of Platelet-to-Neutrophil Ratio in Diabetic Macular Edema, BMC Ophthalmology. (2025) 25, no. 1, 10.1186/s12886-025-04001-2. PMC11966880 40175989

[30] Sipeki N. , Antal-Szalmas P. , Lakatos P. L. , and Papp M. , Immune Dysfunction in Cirrhosis, World Journal of Gastroenterology. (2014) 20, no. 10, 2564–2577, 10.3748/wjg.v20.i10.2564. PMC3949265 24627592

[31] Sanyal A. J. , Anstee Q. M. , Trauner M. et al., Cirrhosis Regression is Associated With Improved Clinical Outcomes in Patients With Nonalcoholic Steatohepatitis, Hepatology. (2022) 75, no. 5, 1235–1246, 10.1002/hep.32204. PMC9303958 34662449

[32] Patel K. and Sebastiani G. , Limitations of Non-Invasive Tests for Assessment of Liver Fibrosis, Journal of Hepatology Reports: Innovation in Hepatology. (2020) 2, 10.1016/j.jhepr.2020.100067. PMC7047178 32118201

[33] Li L. , Huang Q. , Yang L. et al., The Association Between Non-Alcoholic Fatty Liver Disease (NAFLD) and Advanced Fibrosis With Serological Vitamin B12 Markers: Results From the NHANES 1999-2004, Nutrients. (2022) 14, no. 6, 10.3390/nu14061224. PMC8948655 35334881

[34] Graupera I. , Thiele M. , Serra-Burriel M. et al., Low Accuracy of FIB-4 and NAFLD Fibrosis Scores for Screening for Liver Fibrosis in the Population, Clinical Gastroenterology and Hepatology. (2022) 20, no. 11, 2567–2576.e2566, 10.1016/j.cgh.2021.12.034. 34971806

[35] Shi H. , Schweren L. J. , ter Horst R. et al., Low-Grade Inflammation as Mediator Between Diet and Behavioral Disinhibition: A UK Biobank Study, Brain, Behavior, and Immunity. (2022) 106, 100–110, 10.1016/j.bbi.2022.07.165. 35944739

