# Supplementary material for: Association of the Platelet‐to‐Neutrophil Ratio With Metabolic Dysfunction‐Associated Steatotic Liver Disease and Liver Fibrosis in U.S. Adults: NHANES 2017–March 2020
Source: Int J Endocrinol. 2026 Oct 13;2026:5591067. doi: 10.1155/ije/5591067 (PMC13662521; doi:10.1155/ije/5591067)
Supplement: Supplementary file 1 [file IJE-2026-5591067-s001.docx]

**Supplementary Material**

*Association of the Platelet-to-Neutrophil Ratio with Metabolic Dysfunction-Associated Steatotic Liver Disease and Liver Fibrosis in US Adults: NHANES 2017–March 2020*

**Table S1. Operational definitions of covariates and source variables.**

Definitions apply to the analytic sample (N = 3,437). Laboratory variables were measured in the morning fasting subsample. BMI, body mass index; PNR, platelet-to-neutrophil ratio; CAP, controlled attenuation parameter; LSM, liver stiffness measurement.

| **Covariate** | **Operational definition** | **NHANES variable(s)** | **Coding / categorization** |
| --- | --- | --- | --- |
| **Demographics** | | | |
| Age | Age at examination interview, years | RIDAGEYR | Continuous |
| Sex | Self-reported biological sex | RIAGENDR | Male / Female |
| Race/ethnicity | Self-reported race and ethnicity | RIDRETH3 | Mexican American, Other Hispanic, Non-Hispanic White, Non-Hispanic Black, Non-Hispanic Asian, Other/Multiracial |
| Education | Highest level of education attained | DMDEDUC2 | <9th grade, 9–11th grade, High school/GED, Some college/AA, College+ |
| Marital status | Living with a partner or spouse | DMDMARTZ | Married (yes/no) |
| Income-to-poverty ratio | Family income relative to the poverty threshold | INDFMPIR | Continuous |
| **Lifestyle** | | | |
| Smoking status | Never (<100 cigarettes lifetime); current (≥100 cigarettes and smoking now); former (≥100 cigarettes and not smoking now) | SMQ020, SMQ040 | Never / Current / Former |
| Physical activity | Weekly MET-minutes from work, transport, and recreational activities (GPAQ); physically active defined as ≥600 MET-min/week (WHO recommendation) | PAQ605–PAQ675 | Active (yes/no) |
| Alcohol intake | Average number of drinks per drinking day over the past 12 months (1 drink = 14 g ethanol) | ALQ130 | Continuous; heavy use defined as >5 drinks/day (men) or >4 drinks/day (women) |
| **Clinical conditions** | | | |
| Diabetes | Self-reported physician diagnosis, use of glucose-lowering medication, fasting plasma glucose ≥126 mg/dL, or HbA1c ≥6.5% | DIQ010, DIQ070, LBXGLU, LBXGH | Yes / No |
| Hypertension | Self-reported physician diagnosis or use of antihypertensive medication | BPQ020, BPQ040A | Yes / No |
| Dyslipidemia | Use of lipid-lowering medication, triglycerides ≥150 mg/dL, or HDL-C ≤40 mg/dL (men) / ≤50 mg/dL (women) | BPQ050A, LBXTR, LBDHDD | Yes / No |
| **Laboratory biomarkers** | | | |
| ALT | Alanine aminotransferase, U/L | LBXSATSI | Continuous |
| AST | Aspartate aminotransferase, U/L | LBXSASSI | Continuous |
| GGT | Gamma-glutamyltransferase, U/L | LBXSGTSI | Continuous |
| Albumin | Serum albumin, g/dL | LBXSAL | Continuous |
| Total cholesterol | Serum total cholesterol, mg/dL | LBXSCH | Continuous |
| Fasting plasma glucose | mg/dL | LBXGLU | Continuous |
| HbA1c | Glycohemoglobin, % | LBXGH | Continuous |
| Triglycerides | mg/dL | LBXTR | Continuous |
| HDL-C | High-density lipoprotein cholesterol, mg/dL | LBDHDD | Continuous |
| Platelet count | 10³ cells/μL | LBXPLTSI | Continuous |
| Neutrophil count | 10³ cells/μL | LBDNENO | Continuous |
| **Exposure and outcomes** | | | |
| PNR | Platelet count divided by neutrophil count; log2-transformed for regression | Derived from LBXPLTSI, LBDNENO | Continuous (log2-PNR) and quartiles |
| Hepatic steatosis | CAP ≥ 274 dB/m | LUXCAPM | Yes / No |
| MASLD | Hepatic steatosis plus ≥1 of five cardiometabolic criteria per the 2023 SLD nomenclature | Derived | Yes / No |
| Advanced liver fibrosis | LSM ≥ 8.2 kPa | LUXSMED | Yes / No |
| Viral hepatitis exclusion | Current HBV infection: HBsAg positive; current HCV infection: HCV RNA positive | LBDHBG, LBXHCR | Excluded if positive |

**Table S2. Numbers of participants and outcome events by PNR quartile.**

Unweighted counts and weighted percentages (fasting subsample weights). PNR, platelet-to-neutrophil ratio.

| **PNR quartile** | **Unweighted n** | **Weighted population** | **MASLD cases (n)** | **MASLD (weighted %)** | **Advanced fibrosis cases (n)** | **Advanced fibrosis (weighted %)** |
| --- | --- | --- | --- | --- | --- | --- |
| Q1 | 860 | 57,543,253 | 429 | 50.6 | 139 | 14.7 |
| Q2 | 859 | 57,022,947 | 393 | 43.4 | 100 | 9.6 |
| Q3 | 859 | 54,849,286 | 349 | 38.5 | 50 | 4.8 |
| Q4 | 859 | 51,742,581 | 287 | 33.1 | 40 | 3.3 |

*Note: weighted population and weighted case counts reflect fasting subsample weights (WTSAFPRP) scaled to the US civilian noninstitutionalized population; unweighted counts are also shown for transparency (reviewer item 8).*

**Table S3. Complete-case sensitivity analysis (no imputation).**

Participants with any missing Model 4 covariate were excluded (n = 657 excluded; n = 2,780 analyzed), in contrast to the primary analysis using multiple imputation (m = 20, N = 3,437). Models and weighting are identical to the primary analysis.

| **Variable** | **Model 1** | **Model 2** | **Model 3** | **Model 4** |
| --- | --- | --- | --- | --- |
| **MASLD** | | | | |
| log2-PNR (per doubling of PNR) | 0.63 (0.55–0.74) | 0.72 (0.62–0.84) | 0.75 (0.63–0.89) | 1.22 (0.95–1.58) |
| P-value | <0.001 | <0.001 | 0.010 | 0.214 |
| **PNR quartiles (reference: Q1)** |  |  |  |  |
| Q1 | 1.00 (reference) | 1.00 (reference) | 1.00 (reference) | 1.00 (reference) |
| Q2 | 0.72 (0.53–0.98) | 0.80 (0.59–1.09) | 0.78 (0.57–1.07) | 1.01 (0.70–1.43) |
| Q3 | 0.61 (0.47–0.79) | 0.73 (0.56–0.95) | 0.74 (0.55–0.98) | 1.29 (0.86–1.92) |
| Q4 | 0.48 (0.37–0.63) | 0.59 (0.44–0.78) | 0.62 (0.46–0.83) | 1.34 (0.90–1.99) |
| P for trend | <0.001 | <0.001 | 0.010 | 0.193 |
| **Advanced fibrosis** | | | | |
| log2-PNR (per doubling of PNR) | 0.40 (0.30–0.53) | 0.42 (0.30–0.58) | 0.43 (0.30–0.61) | 0.72 (0.48–1.06) |
| P-value | <0.001 | <0.001 | 0.002 | 0.198 |
| **PNR quartiles (reference: Q1)** |  |  |  |  |
| Q1 | 1.00 (reference) | 1.00 (reference) | 1.00 (reference) | 1.00 (reference) |
| Q2 | 0.61 (0.41–0.91) | 0.65 (0.41–1.02) | 0.64 (0.41–1.01) | 0.83 (0.48–1.44) |
| Q3 | 0.32 (0.18–0.55) | 0.35 (0.20–0.60) | 0.36 (0.20–0.64) | 0.66 (0.36–1.20) |
| Q4 | 0.19 (0.11–0.34) | 0.21 (0.11–0.40) | 0.22 (0.12–0.41) | 0.52 (0.26–1.04) |
| P for trend | <0.001 | <0.001 | <0.001 | 0.148 |

*Values are odds ratios (95% confidence intervals) from survey-weighted logistic regression. Model 1: unadjusted. Model 2: age, sex, race/ethnicity. Model 3 (main): plus education, marital status, income-to-poverty ratio, smoking, physical activity. Model 4 (sensitivity): plus body mass index, diabetes, hypertension, dyslipidemia, ALT.*

**Table S4. Sensitivity analysis excluding moderate alcohol intake above the 2023 SLD nomenclature allowance.**

Participants with alcohol intake >2 drinks/day (men) or >1 drink/day (women) were additionally excluded (n = 1,005 excluded; n = 2,432 analyzed; multiple imputation m = 20 applied within the restricted sample). All models and weighting are identical to the primary analysis.

| **Variable** | **Model 1** | **Model 2** | **Model 3** | **Model 4** |
| --- | --- | --- | --- | --- |
| **MASLD** | | | | |
| log2-PNR (per doubling of PNR) | 0.56 (0.44–0.71) | 0.64 (0.50–0.81) | 0.62 (0.49–0.80) | 1.05 (0.79–1.41) |
| P-value | <0.001 | <0.001 | <0.001 | 0.726 |
| **PNR quartiles (reference: Q1)** |  |  |  |  |
| Q1 | 1.00 (reference) | 1.00 (reference) | 1.00 (reference) | 1.00 (reference) |
| Q2 | 0.81 (0.53–1.23) | 0.89 (0.60–1.33) | 0.85 (0.58–1.24) | 1.19 (0.83–1.72) |
| Q3 | 0.56 (0.41–0.77) | 0.67 (0.48–0.93) | 0.61 (0.43–0.86) | 1.19 (0.81–1.75) |
| Q4 | 0.42 (0.28–0.61) | 0.51 (0.35–0.76) | 0.51 (0.35–0.74) | 1.17 (0.76–1.81) |
| **Advanced fibrosis** | | | | |
| log2-PNR (per doubling of PNR) | 0.34 (0.25–0.46) | 0.36 (0.26–0.50) | 0.36 (0.25–0.51) | 0.67 (0.46–0.96) |
| P-value | <0.001 | <0.001 | <0.001 | 0.030 |
| **PNR quartiles (reference: Q1)** |  |  |  |  |
| Q1 | 1.00 (reference) | 1.00 (reference) | 1.00 (reference) | 1.00 (reference) |
| Q2 | 0.55 (0.35–0.87) | 0.57 (0.35–0.92) | 0.57 (0.35–0.91) | 0.77 (0.44–1.38) |
| Q3 | 0.22 (0.12–0.38) | 0.23 (0.14–0.40) | 0.23 (0.14–0.39) | 0.45 (0.25–0.83) |
| Q4 | 0.19 (0.10–0.35) | 0.20 (0.10–0.39) | 0.21 (0.11–0.41) | 0.55 (0.26–1.15) |

*Values are odds ratios (95% confidence intervals) pooled over 20 imputations using Rubin's rules. The 2023 nomenclature allows ≤2 drinks/day in men and ≤1 drink/day in women for the MASLD classification; participants above these levels (i.e., the MetALD/alcohol-associated spectrum) were excluded in this sensitivity analysis. Quartile cut-points were re-derived within the restricted sample.*
